# Leveraging ACL Funding to Implement an Evidence-Based Falls Prevention Program in Three GWEPs

**DOI:** 10.1093/geroni/igab046.1421

**Published:** 2021-12-17

**Authors:** Ellen Flaherty, Nina Tumosa

## Abstract

Primary care practices have a robust capacity to screen older adults for falls risk and refer them to evidence-based falls prevention programs delivered by Community Based Organizations (CBOs). However, due to a difference in the culture and nature of the work done in these two systems of care, there is often a lack of coordination and communication. Dartmouth has worked to bridge this gap for the past five years through our Health Resources and Services Administration (HRSA)-funded Geriatric Workforce Enhancement Program (GWEP). GWEP goals include the promotion of Age-Friendly Health Systems by focusing on the 4 Ms: What Matters Most, Medication, Mentation and Mobility. GWEPs commonly operationalize the Mobility component via falls risk screening and prevention programs. Though CBOs are well suited to deliver falls prevention programs, implementing, disseminating and sustaining community-based falls prevention programs in an environment of cost containment, limited funds for community-based services and workforce issues is challenging. Previous Administration for Community Living (ACL) grant funding enabled us to develop the Dartmouth Falls Prevention Training Center (D-TC) using our expertise in training and community-based implementation of evidence-based interventions. The D-TC offers training and implementation support to primary care and CBOs on screening, referring and capacity-building for falls prevention programs. We will discuss challenges and successes implementing the Dartmouth falls prevention model with two additional GWEP grantees, Baystate and the University of Rhode Island. Benefits of leveraging ACL and HRSA funding to achieve synergistic goals to reduce falls in older adults will be explored.

